# Artificial intelligence on inflammatory dermatoses: where we are and where are we going?^☆^

**DOI:** 10.1016/j.abd.2025.501164

**Published:** 2025-07-18

**Authors:** Dimitri Luz F. Silva, Rafael Rubinho, Ariany Denofre, Sandra Avila, Renata Ferreira Magalhães

**Affiliations:** a Department of Dermatology, Universidade de Santo Amaro, São Paulo, SP, Brazil; b Discipline of Dermatology, Universidade de Campinas, Campinas, SP, Brazil; c Escola de Engenharia Elétrica e de Computação de Campinas, Universidade de Campinas, Campinas, SP, Brazil

**Keywords:** Artificial intelligence, Dermatology, Convolutional neural networks, Hidradenitis suppurativa, Psoriasis

## Abstract

**Background:**

Artificial intelligence (AI) is increasingly gaining ground in dermatology, with studies reporting accuracy equal to or greater than dermatologists for the diagnosis of skin lesions from clinical and dermoscopic images.^1^ AI has been developed and improved constantly for dermatology, however, the focus has been much more on neoplastic diseases, due to their high prevalence and high morbidity.

**Objectives:**

Describe the possible applications of AI in inflammatory dermatoses.

**Methods:**

Articles published between 2013 and 2023 in Medline and Lilacs were retrieved after applying the inclusion and exclusion criteria 19 articles were selected. From each selected article, the necessary information was extracted and with this data, the present review was written.

**Results:**

The first studies on AI in dermatology focused on the diagnosis of neoplasms, especially melanoma, due to the ease of standardization of images, obtaining accuracy equivalent to that of a dermatologist in clinical and dermoscopic lesions. Actually, there are many studies on artificial intelligence in inflammatory dermatosis, such as psoriasis, helping to calculate the PASI, hidradenitis suppurativa, and atopic dematitis.

**Study limitations:**

The limitation of the study is that it is a literature review and because it is an innovative topic with a limited number of studies published in the literature.

**Conclusions:**

Considerable of what is published in the literature is in computer science journals, but it is possible to perceive that there is an important interest in the area and that artificial intelligence will advance to assist dermatologists.

## Introduction

Artificial intelligence (AI) is increasingly gaining ground in dermatology, with studies reporting accuracy equal to or greater than dermatologists for diagnosing skin lesions from clinical and dermoscopic images. However, there is currently no clinical validation in the real world, being mainly used in research protocols.^1^ In the current scenario, AI has three main applications in dermatology: telemedicine, including triage for referral to dermatologists; screening and clinical evaluation applications prior to consultations; and dermatopathology, in order to accelerate diagnosis and assist the pathologist.^2^, ^3^

Studies on the application of convolutional neural networks in dermoscopy for classification algorithms have demonstrated superior performance in the early detection of melanocytic lesions, particularly melanoma. For this purpose, a dataset of 100 dermoscopic images was analyzed, with participants divided into three groups. The first two groups consisted of dermatologists skilled in dermoscopic techniques: Group 1 had access to images only, while Group 2 had access to both images and clinical data. Group 3 employed AI algorithms developed by the authors. Diagnostic specificity reached 82.5% in the AI group, compared to 71.3% and 75.7% in Groups 1 and 2, respectively.^3^, ^4^

AI has been developed and improved constantly for dermatology. However, it focuses mostly on neoplastic diseases, due to their high prevalence and high morbidity. Inflammatory skin diseases, such as psoriasis, are in initial steps, with applications for triage of lesions, with a sensitivity of up to 20% and specificity of 62% only with AI.^3^, ^4^ This review article presents possible applications of AI in inflammatory dermatoses.

## Methods

A narrative review of articles published between 2013 and 2023 in Medline and Lilacs search platforms was conducted using the following keywords: “AI”; “psoriasis”; “hidradenitis suppurativa”; “atopic dermatitis”; “dermatology”, associated with the boolean operators “and” and “or”.

Inclusion criteria for articles: articles in Portuguese or English; published between 2013 and 2022; prospective or retrospective observational studies; systematic and non-systematic literature reviews; cohort studies; clinical trials; addressing the theme “AI and dermatology”.

Exclusion criteria: case reports, case series, editorials, expert opinion, reviews, studies without complete original texts with online access, articles not written in English or Portuguese, and articles that, after selection in the inclusion and exclusion criteria, presented themes not related to AI and dermatology.

In the initial search with keywords described above and Boolean terms, 52 articles were found on the MEDLINE platform and 8 on the LILACS platform. After applying the inclusion and exclusion criteria, 43 articles were found. After applying the criteria 19 articles were selected. It is worth mentioning that application of these criteria was carried out independently by the authors of this dissertation, including only those which was consensus for inclusion.

From each selected article, the main data on AI were selected and included in the present review.

### AI and psoriasis

Psoriasis is an inflammatory disease that affects 125 million people worldwide. Its evaluation can be performed using the Psoriasis Area and Severity Index (PASI), a validated tool most used in patients with plaque psoriasis. PASI is essential so that the dermatologist has a numerical parameter of the patient's evolution, especially in the era of advances in immunobiologicals.^4^

It is observed that PASI presents significant intra- and inter-rater variation, which is calculated in up to 33% of cases, mainly in university centers where the same dermatologist does not evaluate the patient. AI can help both in the diagnosis and calculation of PASI, as well as in the application of personalized protocols and prediction of clinical evolution.^4^, ^5^, ^6^

The first AI program for psoriasis was developed in 2014 to predict the risk of psoriasis in two genetic databases with 99% accuracy. Afterward, Deep Learning programs began to be developed to calculate the PASI, aiming to facilitate and standardize the assessment. Programs are developed based on the opinions of specialists in the area, and image banks are used for training. From this, the AI calculates the score from image analysis, and this result is compared with expert opinion. So far, few published studies have focused on part of the PASI score and not on the final value.^7^

The first evaluation was carried out by Shrivastava et al. from the analysis of each pixel of the photos of the body segments, being classified as a skin or area of the lesion, with an accuracy of 99%. However, it used only 670 images for training, which may have generated an unreliable result. Other studies accurately calculated the PASI subcategories, ranging from 93.8% to 48.8%. There is one that only evaluated erythema and desquamation, with accuracies of 78.9% and 88.7% respectively.^6^, ^8^

**Figure 1:**
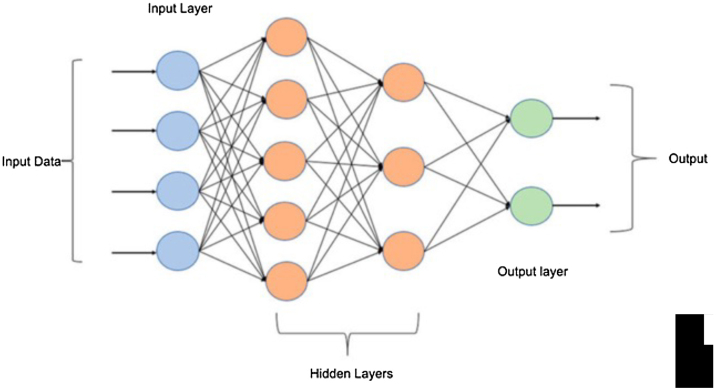
Multilayer perceptron basic architecture.

A recent 2022 publication used 705 trunk photos for Convolutional Neural Network (CNN- an example is demonstrated in Fig. 1) training called Single-Shot PASI. Afterward, the test was validated with 10 images, that were selected as tests sets and were excluded from the training sets. The study also compared the difference in PASI before and after the aid of the AI (the specialist calculated the PASI and then saw the PASI of the AI, being able to redo it), showing that the AI helps a more homogeneous classification among the evaluators. However, it is highlighted that, ideally, training should be performed with more than 10,000 images for deep learning models, but it is possible to perform with less.^9^

Three recent papers obtained positive results with programming for calculating the complete PASI: Huang et al. used 1962 patients for training and 405 to validate the test. The model was compared with the evaluation of 43 experienced dermatologists. Finally, the model was incorporated into an application in which patients could take a picture of themselves to include the PASI in the medical record.^10^

Another important work was carried out with Schaap et al., training the CNN with 576 images of the back, 614 of the arms, and 541 of the legs, using the classification of specialists as a basis and then compared with the evaluation of only the image of the trunk with 5 physicians with PASI experience. The AI had a similar performance, with a better assessment of the body surface area, reaching an accuracy of 79.4%. However, the comparison of total PASI was not evaluated, and comparisons of more severe scores were excluded due to a lack of sufficient images for training.^11^

Finally, Fink et al. obtained a concordance of 0.86 in evaluating 120 patients, comparing AI and experienced physicians. The agreement remained high when the evaluation was repeated, which did not occur with physicians. A previous study at the institution showed a difference of 27% in the calculation of PASI. When repeating the same patient after 4-weeks, there was a variation of up to 33%. Photos were captured using FotoFinder with 4 predefined poses, standardizing the method.^12^

As previously mentioned, an important limitation of Deep Learning is that a large number of images are required for training and reliable validation of the method. In addition, most of the published studies were carried out with the same ethnic group, which makes them little applicable worldwide so far. There is also a limitation in evaluating curved areas, such as elbows and knees, typically affected by psoriasis.

### AI and atopic dermatitis

Atopic dermatitis is the most common chronic inflammatory skin disease in the world, with an essential impact on the quality of life of patients, productivity at work, and health systems, with an approximate worldwide prevalence of 15% to 28%, being of multifactorial origin and complex pathophysiology. Dermatology already benefits from AI in several areas, which has proved to be fundamental as an adjuvant method for diagnosis, classification of severity, and personalization of care, as well as an adjuvant for defining therapy in dermatoses, including, albeit incipient, in atopic dermatitis. However, few publications in the area demonstrate a potential for development and research today.^6^

A study carried out by Guimarães and collaborators in 2021 proposed an AI algorithm for CNN-based analysis of tomographic images of patients with atopic dermatitis (AD). In atopic dermatitis, it is possible to identify changes such as cellular irregularity, intracellular changes, perinuclear accumulation of mitochondria, and various metabolic changes. Despite the low sample size, this study demonstrated high sensitivity and specificity for diagnosing AD according to the imaging characteristics, thus providing a new method with increased diagnostic accuracy.^13^, ^14^

Due to the multifactorial origin of atopic dermatitis and the correlation of genetic mutations and alterations in the intestinal microbiome as predisposing factors for the development of AD, Jiang et al. published a study in 2022 in which they described machine learning to predict the risk of developing AD. AD and the phenotypic profile according to these factors. This controlled study captured 161 patients and evaluated 35 genes and 50 microbial characteristics, and with limitations of the study, they published an algorithm capable of accurately differentiating based only on transcriptome and microbiome data from healthy individuals affected by AD.^15^

Atopic dermatitis currently has consolidated scores in the literature, such as the SCORAD (“SCORing Atopic Dermatitis) and EASI (Eczema Area and Severity Index). However, numerous variables depend on examiners in SCORAD, presenting different classifications in the same patients when applied by different experienced dermatologists. A study carried out by Medela and collaborators created an AI algorithm capable of classifying and calculating the SCORAD through the interpretation of patient images in a fast, objective, and fully automated way. This new model would have an impact both in clinical practice by optimizing the time for calculating the SCORAD, subjectivity in the scores, and allowing classification in virtual consultations, as well as in clinical trials, to the point that it would reduce the SCORAD classification biases by the examiners.^16^

Some studies sought to develop CNN to automate the EASI classification, developing AI capable of measuring the degree of erythema, papules, excoriation, and lichenification by capturing images of patients. A study carried out in 2021 by Bang and collaborators captured 8000 clinical images of atopic dermatitis without photo quality criteria, inserted them into four different CNN platforms to allow automated EASI calculation, and demonstrated classification rates very similar to those attributed by the professional dermatologist demonstrating the effectiveness of AI and the possibility of its clinical applicability.^17^

Finally, it is noted that the role of AI is still incipient in atopic dermatitis; however, published algorithms and prototypes, ranging from methods for diagnosis, classification and, consequently, influence on the therapeutic definition, have shown a promising space for both clinical practice daily, as in clinical trials and other scientific studies.^15^, ^16^

### AI and hidradenitis suppurativa

Hidradenitis suppurativa (HS) is a chronic inflammatory skin condition that causes nodules, abscesses, and fistulas in the armpits, groin, and other areas of skin folds. HS is a debilitating disease that can significantly affect patients' quality of life, with cumulative impacts throughout an individual's lifetime.^18^

AI is a technology that can be used to improve the diagnosis, treatment, and prognosis of HS, as well as new diagnostic methods (use of medical images to identify HS lesions), develop new treatments (immunotherapy and gene therapy) or even in the proper screening of patients, as in a work that used a chatbot and patients imputed their symptoms, later being evaluated for a more accurate diagnosis.^18^, ^19^

AI can also be used to improve the prognosis of HS, predict the risk of complications, and develop rehabilitation programs for patients with HS. To standardize the classification of this disease, a Spanish group tested the AI in the evaluation and application of the International Hidradenitis Suppurativa Severity Score System (IHS4), a disease severity score used both in practice and in clinical studies, which may mean, in addition to a better classification of the disease, to better predict prognosis and which best treatment should be instituted at the best stage of the disease. The work even shows that the AI model used had comparable results with the clinical evaluation of experts, according to the consensus and standards already studied in the literature, exemplifying an AI performance model applicable to the routine of dermatologists who treat HS.^20^

### AI and other dermatosis

The first studies on AI in dermatology focused on diagnosing neoplasms, especially melanoma, due to the ease of standardization of images, obtaining accuracy equivalent to dermatologists. As a justification, it can be used in teleconsultation to improve patient access to specialized care.

**Figure 2:**
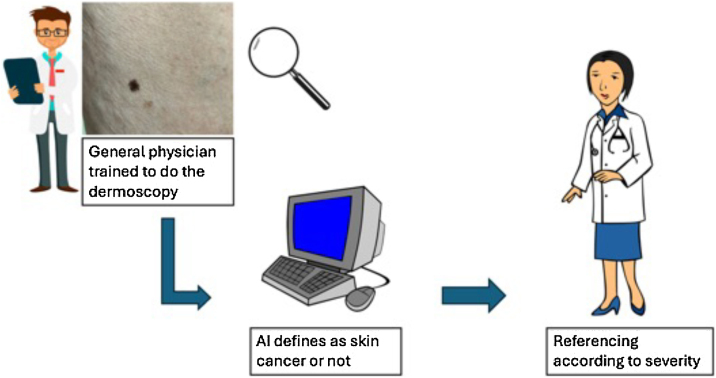
How can AI improve the skin cancer diagnosis? – Naevus on the left leg of a 50 year-old patient (UNICAMP archive).

About half of skin-related visits are with non-dermatologists, who lack specialized training. When comparing the AI diagnosis with the primary care doctor, the AI performed better. In 2016, the first program diagnosed melanoma using 170 images of melanoma and 170 of nevi with a sensitivity and specificity of approximately 0.8. It was only in 2017, with the use of CNN, that it was possible to perform the differential diagnosis between melanoma and benign nevus and epithelial tumors and seborrheic keratosis. In studies, CNN's performance is comparable to that of an experienced dermatologist, or even better^3^, ^4^ With the use of IA, it is possible to accelerate the diagnosis of skin cancer by a general physician, after training in the use of dermoscopy, so that the referral can be as quick as severity demands (Fig. 2).

The use of AI in histopathology is also possible; there is good performance reported in the diagnoses of BCC, prostate, and breast neoplasms as well. It is possible to increase accuracy up to 78%, mainly if molecular analysis is used for training. The method analyzes each pixel of the image as malignant or not in a binary way. A significant advantage would be access to diagnosis in places with no dermatopathologist.^1^, ^2^

It is essential to highlight that most of the models were created in Europe and East Asia, with essential limitations for use in other populations as well. Another critical factor is that the dermatologist analyzes other factors to consider malignancy in diagnosing melanoma, while AI cannot, leading to more false positives. The use of AI also requires standardization of photos with quality and good lighting, something difficult to obtain on a large scale. In addition, there is no study with real-world experience or with rarer lesions such as amelanotic melanoma. The dermatologist diagnoses more than 2000 skin diseases, while the algorithm was trained for up to 757 diseases, with binary classification as malignant vs. benign. It does not perform well when used to distinguish diagnoses.^1^, ^2^, ^3^

## Conclusion

The integration of AI in dermatology will occur as research advances. With the perspective to enhance the accuracy of diagnosis and follow up besides the availability of specialist diagnosis for skin cancer and inflammatory skin diseases, AI can help to speed patient access to treatment, especially in places where dermatologist is not easily available. The need of a large dataset for algorithm training is still the major difficulty for validation however, good evidence with high accuracy has been shown. Another important highlight is that most algorithms are trained with white and Asian skin, limiting the application for mixed-race populations, like Brazil. Future research will be crucial to overcoming these barriers and realizing the full potential of AI in dermatology (Table 1).

**Table 1:** Summary of the main advances in AI in dermatology.

Subtheme	Authors	Key Data
AI and Psoriasis	Shrivastava S, et al.	AI analysis of body segment photos for psoriasis with 99% accuracy. Study used 670 images for training.
	Huang J, et al.	Deep learning model for PASI calculation using 1962 patients for training and 405 for validation. Accuracy achieved.
	Schaap T, et al.	CNN trained with 576 images of the back, 614 of the arms, and 541 of the legs, achieving 79.4% accuracy in body surface area assessment.
	Fink C, et al.	AI concordance of 0.86 in PASI evaluation of 120 patients. High agreement with experienced physicians. Excluded curved body areas and areas covered by hair.
AI and Atopic Dermatitis	Guimarães TA, et al.	AI algorithm for CNN-based analysis of multiphoton tomography images for atopic dermatitis. High sensitivity and specificity for diagnosis.
	Jiang Q, et al.	Machine learning to predict risk of developing atopic dermatitis based on 35 genes and 50 microbial characteristics. Accurate differentiation of AD.
	Medela A, et al.	AI algorithm for automated SCORAD classification from patient images. Optimizes calculation time and reduces subjectivity.
	Bang Y, et al.	Automated EASI calculation using CNN with 8000 clinical images Classification rates similar to professional dermatologists.
AI and Hidradenitis Suppurativa	Hernández I, et al.	AIHS4 uses Legit.Health-IHS4net based on YOLOv5 architecture. Assesses HS severity with performance comparable to expert physicians. Reduces inter-observer variability.
	Wiala A, et al.	Uses CNNs for HS severity classification. Dataset includes 7675 images. Overall prediction accuracy of 78% and IHS4 classification accuracy of 72%. U-NET algorithm localizes lesions with 88.1%-pixel accuracy.
	Crovella S, et al.	Integrates AI for early HS diagnosis using machine learning and deep learning algorithms for image analysis. Recognizes subtle patterns and addresses challenges like data privacy.
AI and Other Dermatoses	Young AT, et al.	Review of AI applications in dermatology, including telemedicine screening, and dermatopathology. Highlights CNNs’ performance in diagnosing melanocytic lesions and AI’s role in PASI calculations HS management, and onychomycosis diagnosis.

CNN, Convolutional Neural Network; AT, Atopic Dermatitis; EASI, Eczema Area and Severity Index; HS, Hidradenitis Suppurativa; AI, Artificial Intelligence; PASI, Psoriasis Area and Severity Index; SCORAD, SCORing Atopic Dermatitis.

## Financial support

None declared.

## Authors' contributions

Dimitri Luz Felipe da Silva: Drafting and editing of the manuscript.

Rafael Rubinho: Drafting and editing of the manuscript.

Ariany Denofre: Drafting and editing of the manuscript.

Sandra Avila: Review of the manuscript.

Renata Ferreira Magalhaes: Review and approval of the final version of the manuscript.

## Conflicts of interest

None declared.
